# Tetraspanin CD81 is an adverse prognostic marker in acute myeloid leukemia

**DOI:** 10.18632/oncotarget.11481

**Published:** 2016-08-22

**Authors:** Thomas Boyer, Soizic Guihard, Christophe Roumier, Pauline Peyrouze, Fanny Gonzales, Céline Berthon, Bruno Quesnel, Claude Preudhomme, Hélène Behal, Alain Duhamel, Catherine Roche-Lestienne, Meyling Cheok

**Affiliations:** ^1^ Hematology Laboratory, Biology and Pathology Center, CHRU Lille, France; ^2^ Jean-Pierre AUBERT Research Center, UMR-S1172, Lille, France; ^3^ Department of Hematology, Claude Huriez Hospital, CHRU Lille, France; ^4^ Institute of Medical Genetics, Jeanne de Flandre Hospital, CHRU Lille, France; ^5^ Department of Biostatistics, Univ. Lille, CHU Lille, EA 2694 - Santé Publique: Épidémiologie et Qualité des Soins, F-59000 Lille, France

**Keywords:** acute myeloid leukemia, prognosis, CD81, tetraspanin, flow cytometry

## Abstract

CD81 is a cell surface protein which belongs to the tetraspanin family. While in multiple myeloma its expression on plasma cells is associated with worse prognosis, this has not yet been explored in acute myeloid leukemia (AML). We measured membrane expression of CD81 on AML cells at diagnosis, evaluated its association with AML characteristics and its influence on patient outcome after intensive chemotherapy in a cohort of 134 patients. CD81 was detected in 92/134 (69%) patients. Patients with AML expressing CD81 had elevated leukocyte count (P=0.02) and were more likely classified as intermediate or adverse-risk by cytogenetics (P<0.001). CD81 expression had a negative impact on survival (event-free survival, overall survival and relapse-free survival) in univariate (P<0.001) and in multivariate analyses (P=0.003, 0.002 and <0.001, respectively). CD81 has a negative impact on OS in patients with *NPM1* mutation (P=0.01) and in patients ELN-favorable (P=0.002). In conclusion, this cell surface marker may be a new prognostic marker for diagnostic risk classification and a potential therapeutic target for drug development in AML.

## INTRODUCTION

CD81 antigen belongs to the tetraspanin family (33 members in mammals), which are cell surface transmembrane proteins. This antigen was originally discovered as a target of an antiproliferative antibody and subsequently named TAPA-1 [1]. It associates with other proteins in dynamic membrane entities called tetraspanin-enriched microdomains (TEMs) and partners may vary according to cell type (e.g., CD19 in B cells) [2]. Various cellular functions are linked to CD81 (i.e., BCR signaling in B cells [3], B-T cell interaction [4] and cell entry receptor function for different infectious diseases [5]). Furthermore, recent studies showed that tetraspanins are implicated at several stages of carcinogenesis as well as in metastasis and angiogenesis [6]. Interestingly, Vences-Catalàn and colleagues have demonstrated a dominant role of CD81 affecting metastasis and immunomodulation in cancer [7]. Targeting of CD81 may decrease fusion of metastatic colon carcinoma cells and may improve sensitivity to chemotherapeutic agents [8]. Specifically, CD81 may be important in hematopoiesis as it allows hematopoietic stem cells to re-enter to quiescence [9]. In hematologic malignancies, CD81 has mostly been studied in multiple myeloma where its expression on plasma cells is associated with worse progression free survival (PFS) and overall survival (OS) [10]. Yet, the prognostic value of CD81 has not been addressed in myeloid malignancies, such as AML.

AML is a leading cause of leukemia-related mortality, characterized by maturation arrest and subsequent accumulation of blast cells at various stages of incomplete differentiation, and by reduced production of healthy hematopoietic elements [11]. Importantly, AML is a heterogeneous disease at both the phenotypic and molecular level with a variety of distinct genetic alterations giving rise to the disease. Currently, the combination of three days of daunorubicin and seven days of cytarabine is still accepted as the cornerstone of induction treatment allowing complete remission in younger patients in 70-80% of the cases [12]. Nevertheless, relapse still occurs in approximately half of the patients diagnosed with AML and the 5-year overall survival rate is only about 40% [11].

Over the past few years, identification of new prognostic remains important; especially those potentially refining therapeutic options. The development of prognostic markers is particularly important in AML with normal cytogenetics (CN AML) and currently, three molecular markers (*NPM1-* and *CEBPA* mutations and *FLT3* internal tandem duplication (*FLT3-ITD*)) are used in clinical practice [13]. Prognostic value of CD81 expression in multiple myeloma and its use as a marker in minimal residual disease (MRD) in chronic lymphocytic leukemia (CLL) are well established [14]. In this study, we analyzed the association of CD81 with other biological factors and its effects on patient outcomes in AML.

## RESULTS

### CD81 cell surface expression on normal and AML blast cells

**Figure 1:**
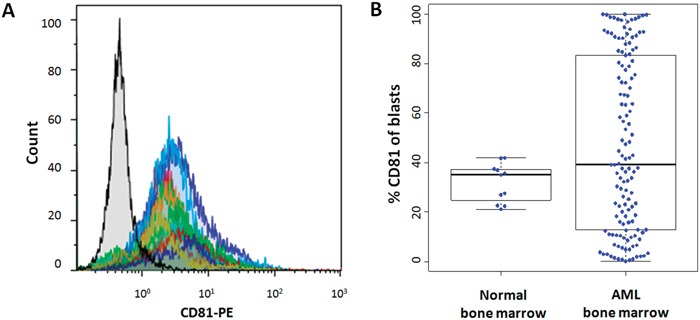
CD81 expression on normal and AML blast cells **A.** Normal bone marrow sample overlay of mean fluorescence intensity histograms of CD81 on blast cells. Isotype control is colored in black. **B.** Comparison of CD81 expression on blast cells between normal bone marrow samples (n=11) and diagnostic bone marrow from patients with de novo AML (n=134).

**Figure 2:**
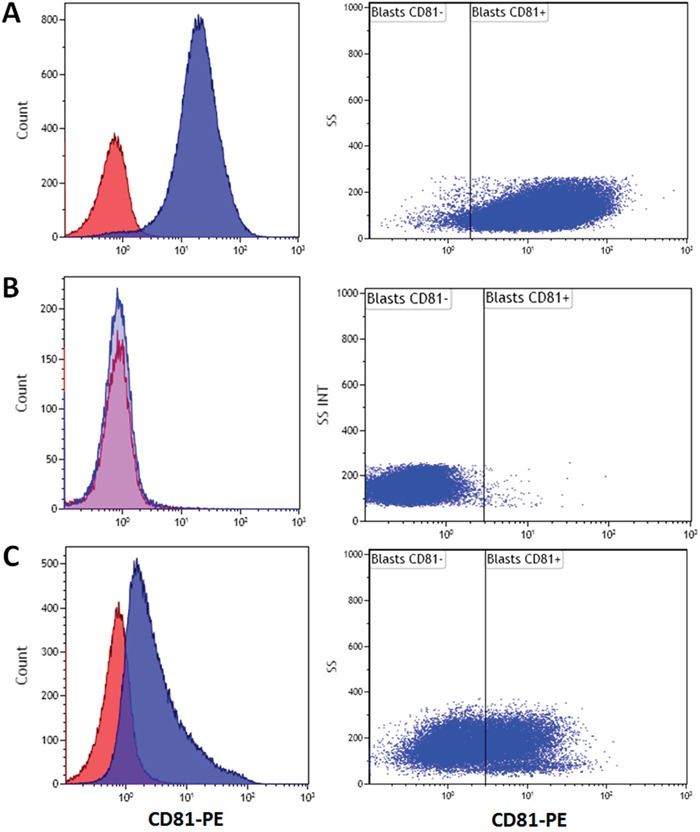
Primary AML have varying CD81 expression on blast cells Representative examples of mean fluorescence intensity histograms of different types of AML according to CD81 expression: **A.** Example of high CD81 blast expression (CD81^++^) **B.** Example of negative CD81 blast expression (CD81^−^); **C.** intermediate CD81 blast expression (CD81^+^). Isotype control corresponds to the red histogram.

CD81 is homogenously expressed on physiologic myeloblasts in normal bone marrows (BM) (mean=32%; range=21 to 42%; n=11; Figure 1A). In contrast, expression of CD81 on AML blasts is more heterogeneous (mean=range=0.1 to 100%, n=134) and significantly higher (47%; P<0.001; Figure 1B). Interestingly, we observed two types of AML firstly, 43% of AML with high CD81 expression (more than 50% of blasts, Figure 2A) and secondly, 31% of AML had no CD81 expression commonly defined as less than 20% of blasts [15] (Figure 2B). While all physiologic myeloblasts showed intermediate CD81 expression, only 25% of AML were found in that range (Figure 2C).

### Association of CD81 with prognostic factors in AML

A total of 134 patients were included in our study with ages ranging from 18 to 78 years. We compared patient characteristics between AML blasts positive vs. negative for CD81 expression (Table 1). Expression of CD81 was found in the majority of AML (92 of 134, 69%), but no CD81 expression was associated with favorable characteristics (i.e., younger age, lower WBC and favorable cytogenetics). In contrast, positive CD81 expression was associated with FAB types M1 and M5 and unfavorable cytogenetics (Table 1). No difference was found for sex, hemoglobin level, platelet count, *FLT3-ITD* and *NPM1* mutational status. CD81 positive AML tended to include more AML with *FLT3-ITD* mutation (P=0.06). AML patients with CD81 positive blast cells were of higher age, had higher white blood cell counts (WBC) at diagnosis (P=0.02) and were more likely to have AML with intermediate or adverse-risk cytogenetics (P<0.001).

**Table 1:** Patient characteristics

	All patients (n=134)	< 20% CD81^−^ Blasts (n=42)	> 20% CD81^+^ Blasts (n=92)	P-value
Gender [M/F]	79/56	24/18	54/38	0.83
Age [years]^$^	51.1±16.0	46.9±16.4	53.0±15.5	**0.04**
WBC [G/L]*	28 (1-325)	14 (2-198)	45(1-325)	**0.02**
FAB type				**<0.0001**
M0	3% (4/101)	3% (1/35)	4% (3/66)	
M1	22% (22/101)	14% (5/35)	26% (17/66)	
M2	28% (28/101)	49% (17/35)	17% (11/66)	
M4	26% (26/101)	31% (11/35)	23% (15/66)	
M5	19% (19/101)	3% (1/35)	27% (18/66)	
M6	2% (2/101)	0% (0/35)	3% (2/66)	
Hemoglobin level [g/dL]^$^	9.5±2.2	9.5±2.3	9.4±2.1	0.81
Platelet count [G/L]*	59 (7-864)	70 (12-864)	58 (7-670)	0.96
Cytogenetic risk, (n/N)				**<0.0001**
Favorable	11% (14/131)	28% (12/42)	2% (2/89)	
Intermediate	75% (98/131)	55% (23/42)	84% (75/89)	
Unfavorable	14% (19/131)	17% (7/42)	14% (12/89)	
*FLT3-ITD*, (n/N)	28% (36/129)	17% (7/41)	33% (29/88)	0.06
*NPM1 mut*, (n/N)	34% (43/125)	28% (10/36)	37% (33/89)	0.32

* median with range in parenthesis;

$ mean ± SD: standard deviation;

WBC: white blood cell count;

M: male; F: female; NA: not applicable.

### Relevance of CD81 as a prognostic marker in AML

At the time of analysis, we counted 40 uncensored deaths and median overall survival was 3.5 years. As expected, unfavorable cytogenetics was associated with poorer OS (hazard ratio [HR]=2.75, 95% confidence interval [CI]=1.48-5.09, P=0.006). *NPM1* mutations were significantly associated with better survival (HR=0.61, 95%CI=0.31-1.20, P=0.03) and *FLT3-ITD* mutation showed no impact on OS (P=0.40).

CD81 expression on blast cells negatively affected EFS, OS and RFS (P<0.001). Multivariate analysis validated the worse prognosis of AML with CD81 expression on EFS, OS and RFS (Table 2).

**Table 2:** Hazard ratio of CD81 adjusted on cytogenetic risk, age at diagnosis and *NPM1*+/*FLT3-ITD*- status

	HR (95% CI)	*P*-value
**EFS**	3.45 (1.5-7.8)	**0.003**
**OS**	4.14 (1.69-10.14)	**0.002**
**RFS**	9.46 (2.66-33.71)	**0.0005**

**Figure 3:**
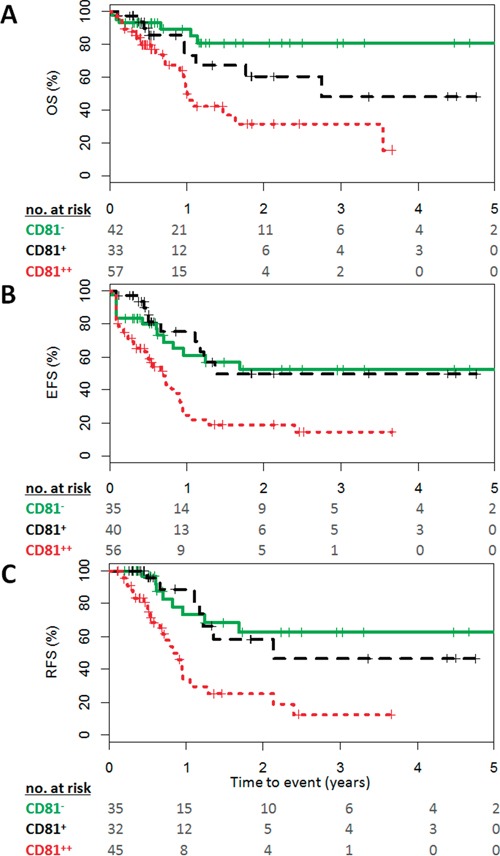
High CD81 expression on blast cells predicts poor outcome in AML Survival curves of **A.** OS, **B.** EFS, **C.** RFS stratified by CD81 expression measured in diagnostic bone marrow of AML patients. Shown is the survival of patients with AML either CD81^−^ in green (less than 20%), CD81^+^ in black (20 to 50%), or CD81^++^ in red (greater than 50%). Numbers at risk at each year of follow-up are given. P-values based on logrank test.

CD81 expression on physiologic myeloblasts in normal BM did not exceed 40% (Figure 1B). Furthermore, CD81 expression on AML blast cells showed a bimodal distribution with a mean of 47%. Thus, we determined three groups of patients based upon CD81 expression on blast cells: CD81^−^ (<20%, n=42), CD81^+^ (20 to 50%, n=34) and CD81^++^ (>50%, n=59). A worse survival was associated with a higher expression of CD81 considering OS, EFS and RFS (P<0.001) compared to those CD81-low (Figure 3).

**Figure 4:**
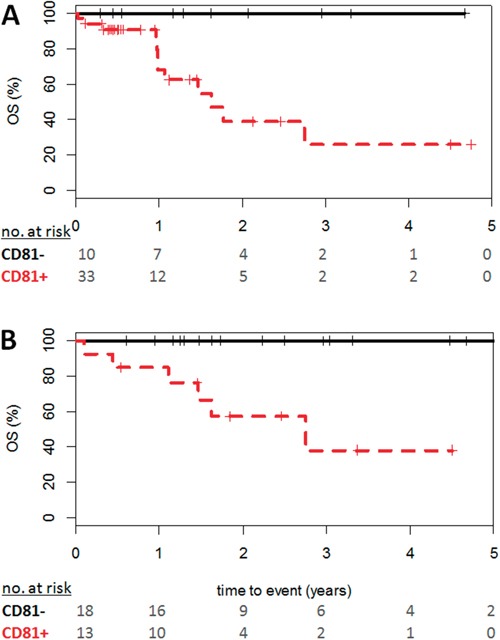
Effect of CD81 expression on Overall Survival in favorable-risk patients Curve shown in black illustrates overall survival of patients with CD81^−^ AML (less than 20%), CD81^+^ in red (greater than 20%) for **A.** *NPM1* mutated patients and **B.** ELN-favorable risk group. Numbers at risk at each year of follow-up are given. P-values based on logrank test.

Interestingly, among the *NPM1* mutated patients, blasts CD81 greater than 20% showed significant inferior OS (P=0.01, Figure 4A) but had no impact on EFS (P=0.24) or RFS (P=0.22). Considering ELN risk categories, subset analyses revealed a significant prognosis impact of CD81 expression for OS in ELN-favorable patients (P=0.002, Figure 4B) whereas no impact was found in other categories.

## DISCUSSION

AML is a heterogeneous disease and prognostic factors have become increasingly important in order to propose appropriate therapy. Currently, cytogenetic analysis is most important for AML risk classification [11] and, according to standard recommendations three risk groups are defined: favorable, intermediate and adverse [16]. In CN AML, prognostic tools are particularly relevant and subgroups have been defined, based on the mutational status of genes such as *FLT3-ITD*, *NPM1*, and *CEBPA* [17]. Nevertheless, new prognostic factors are needed to better discriminate patients with AML. In this study, we evaluated the prognostic impact of CD81 expression in a cohort of 134 adult patients treated with intensive chemotherapy for AML. We found expression of CD81 in 69% of all AML cases and with a higher frequency in AML with *FLT3-ITD* mutation (P=0.06). Expression of CD81 was associated with a worse clinical outcome as it negatively affected survival (EFS, OS and RFS) in univariate and multivariate analyses. Furthermore, this negative impact is even stronger with high CD81 expressing blast cells.

In multiple myeloma, detection of CD81 positive plasma cells was an independent negative prognostic factor for PFS and OS [10]. This study by Paiva et al investigated CD81 expression by MFC in 230 patients with plasma cell myeloma (PCM) and found a positive expression in 45% of the patients. The adverse impact of CD81 was then validated in an additional 325 transplant-candidate PCM patients. Moreover, dim or negative CD81 expression was only observed in abnormal plasma cells [18]. In another study, circulating plasma cells showed significant down-regulation of integrins and activating molecules including CD81; this finding suggests a potential role in plasma cell homing for CD81 [10].

AML with more than 20% of CD81 positive blast cells showed a significant adverse prognosis for EFS, OS and RFS. In our study, these patients were older, had higher white blood count and showed an association with intermediate and adverse-risk cytogenetics compared to patients with less than 20% CD81 blasts. In ELN-favorable group, a percentage of CD81 over 20% had a significant negative impact on OS (P=0.002): this data is particularly interesting as it allows discriminating patients in this low-risk group. Further studies are needed to determine whether this subgroup of patients with AML will benefit from dose-intensified chemotherapy.

CD81 definitely exerts a negative impact on survival outcome in AML patients. Nevertheless, the mechanisms by which CD81 induces poor prognosis in AML are yet unknown. However, as we did not find any difference between patients with CD81^+^ vs. CD81^−^ AML in achieving complete remission (CR) after remission induction therapy, the mechanism may be less likely to be chemotherapy resistance induced by CD81 expression on leukemic blasts. Though, we did note a trend for higher CD81 expression in AML of patients who did not achieve CR. Furthermore, larger cohort of patients is necessary to prove significance of CD81 expression on relapse within subgroups of AML. Furthermore, patients with CD81 positive expression presented with higher WBC at diagnosis, which may be explained by a defect in blast cell homing. Therefore, the role of CD81 in blast cell homing needs to be determined in AML, as similar effects have been described in multiple myeloma [10]. CD81 is physiologically implicated in the re-entry of hematopoietic stem cells into the quiescent state in order to control self renewal after induced proliferation. In leukemic blasts however, any alteration of this function may influence tumor dormancy and treatment outcome in patients with AML with different levels of CD81 expression.

Finally, our study provided the rational for novel therapeutic approaches targeting CD81 to be considered. Accordingly, anti-CD81 have demonstrated in vivo efficacy in HCV [19] and Plasmodium falciparum [20] infections. Future independent studies are needed to confirm prognostic impact of CD81 in AML.

## MATERIALS AND METHODS

### Patients

One hundred and thirty four patients with AML treated by intensive chemotherapy were included in this study. All patients were treated in the department of hematology of Lille hospital. Signed informed consent was obtained from each patient in accordance with the declaration of Helsinki. Cytogenetic risk was determined according to standard criteria [21].

Complete remission (CR) criteria were defined in agreement with the European Leukemia Net recommendation [13].

### Multiparameter flow cytometry (MFC)

Diagnostic blast cells were obtained from fresh or thawed cryopreserved BM aspirates after red blood cell lysis. Of note, MFC results were not different between fresh and frozen BM cells done for five patients (data not shown). Each sample was washed twice with phosphate buffered saline and stained separately for 30 min at room temperature with two antibody panels. The first antibody panel contained: anti-CD36-FITC (clone FA6-152, Iotest, Beckman Coulter Inc., Brea, CA, USA), anti-CD81-PE (clone JS24, Beckman Coulter), anti-CD33-PC5.5 (clone D3HL60.251, Iotest, Beckman Coulter), anti-CD34-AA700 (clone 581, Iotest, Beckman Coulter), and anti-CD45-KO (clone J.33, Iotest, Beckman Coulter).

The second antibody panel included: anti-CD7-FITC (clone 8H8.1, Iotest, Beckman Coulter), anti-CD13-PE (clone SJ1D1, Iotest, Beckman Coulter), anti-CD19-ECD (clone J3-119, Iotest, Beckman Coulter), anti-CD33 PC5.5 (clone D3HL60.251, Iotest, Beckman Coulter), anti-CD117-APC (clone 104D2D1, Iotest, Beckman Coulter), anti-CD34-AA700 (clone 581, Iotest, Beckman Coulter), anti-HLA-DR-PB (clone Immu-357, Iotest, Beckman Coulter), and anti-CD45-KO (clone J.33, Iotest, Beckman Coulter). A minimum of 5 × 10^5^ events were acquired.

Blast cells were gated as CD45^dim^, SSC^low^, CD33^+^ and lymphocytes (CD45^bright^, SSC^low^, CD33^−^), monocytes (CD45^int/bright^, SSC^int^, CD33^bright^) and mature myelomonocytic cells (CD45^int^, SSC^high^, CD33^dim/neg^) were excluded.

Isotype control (clone 7T4-IF5, Iotest, Beckman Coulter) was used to better define the threshold of CD81 positive-stained cells. Results are reported as percent of positive blast cells. If more than 20% of the blast population is stained, the AML sample is considered CD81 positive [22].

Measurements were performed on a Navios flow cytometer and analyzed with Kaluza software (Beckman-Coulter). The cytometer settings were daily tested for optical alignment, fluidic stability, optical detector sensitivity and standardization using adapted fluorospheres (Flowset targets™ and Flowcheck™, Beckman-Coulter) [22].

### Statistical analysis

The distribution of quantitative variables was verified graphically and by a Shapiro-Wilk test and the comparison between normal and AML BM sample on CD81 expression was tested using the Student t-test. Differences between patients with less than 20% of expression of CD81 and those with more than 20% of expression of CD81 on baseline quantitative variables were assessed by Student t- or Mann-Whitney U-test and qualitative variables were compared using Chi-square or Fisher Exact test.

Quantitative variables associated with either overall survival (OS), event-free survival (EFS) or relapse-free survival (RFS) were tested with the Cox model. For patients who underwent bone marrow transplantation, survival was censored at the date of transplantation, and for patients alive, survival was censored at the date of last known alive. The association between expression of CD81 and OS, EFS and RFS was adjusted by cytogenetic risk, age at diagnosis and *NPM1*+/*FLT3-ITD*- status. OS, EFS and RFS were described by the Kaplan Meier method stratified by expression of CD81 according to greater than or equal to 20%, commonly defined as the bottom detection limit [15] and greater than or equal to 50%, defined as CD81 over-expressing AML (i.e., top quartile).

All statistical tests were two-tailed and the significance level was set to 0.05. Statistical analysis was performed with SAS software version 9.3 (SAS Institute, Cary, NC, USA).
